# The Rejuvenating Potential of Plasticizers on Oxidatively Aged Asphalts: Rheological and Molecular Dynamics Perspectives

**DOI:** 10.3390/polym14214624

**Published:** 2022-10-31

**Authors:** Wei Cao, Xinyan Li

**Affiliations:** School of Civil Engineering, Central South University, Changsha 410075, China

**Keywords:** asphalt rejuvenators, pavement sustainability, performance restoration, molecular interactions, asphaltene deagglomeration

## Abstract

Recycle and reuse of waste asphalt materials in the pavement industry has brought tremendous contributions to the infrastructure sustainability and environmental preservation. The recent literature has suggested a great potential of plasticizers to be used for rejuvenating the oxidated paving asphalts. This study was aimed at assessing the rejuvenating effectiveness by rheological characterizations of two typical plasticizers, dibutyl phthalate (DBP) and tributyl citrate (TBC), selected based on the molecular structural differences. The underlying rejuvenating mechanisms were approached using molecular dynamics (MD) simulation, for probing the interactions between the plasticizers and oxidized asphaltenes and examining the outcomes in terms of deagglomeration. The results indicated that both plasticizers were highly effective in restoring the stiffness and elasticity properties as well as fatigue resistance of the aged asphalt. According to the simulations, the two plasticizers were able to deagglomerate the asphaltene associations. Owing to the high polarity and hydroxyl group, TBC appeared to be slightly more efficient in dissociating the asphaltenes, which explained its higher effectiveness in restoring the rheological properties as compared to DBP. Both the rheology and simulation results suggested that the plasticizers were rejuvenating instead of simply softening the aged asphalt.

## 1. Introduction

Asphalt pavement is a resource-intensive infrastructure, due to its tremendous requirements for mineral aggregate as the load bearing skeleton and petroleum asphalt the binding cement. Nowadays, this industry is faced with stricter regulations on environmental protection and resource preservation. Furthermore, the development and implementation of the coking technologies has allowed refineries to reduce the asphalt yield, which raises the cost of asphalt independent of the crude oil [1]. Fortunately, researchers and the industry have developed various means to cope with this situation by reusing waste asphalt materials, such as reclaimed asphalt pavement (RAP) and recycled asphalt shingles (RAS), through the application of rejuvenators and/or warm-mix technologies [2], for instance.

Despite the enormous environmental and economic benefits, the primary challenge to reuse these waste asphalt materials lies in the fact that the asphalt cement has been significantly oxidized. Incorporation of the aged asphalt has been shown to benefit the rutting performance of asphalt pavement. However, the ductility and stress relaxation properties tend to be negatively impacted, leading to pre-mature failures due to fatigue or low-temperature cracking [3,4]. From the microscopic perspective, Petersen et al. [5,6,7] conducted a systematic investigation into asphalt aging and proposed a dual mechanism: the first stage characterized by a fast reaction due to the formation of sulfoxides and the second by a slow and long-term reaction with the formation of ketones and alcohols. This two-stage mechanism and the associated primary oxidative products were later confirmed in a molecular simulation equipped with a reactive force field [8]. 

An immediate consequence of oxidation is an increase in the heavy components, typified by asphaltenes, at the cost of light fractions, such as saturates and aromatics [9]. Rejuvenators, derived primarily from petroleum and bio-oils, contain one or a few components that may be classified by solubility as the SARA (saturates, aromatics, resins, and asphaltenes) family similar with the paving asphalts [10]. Rejuvenators usually feature a high proportion of light molecules that diffuse into the aged asphalts due to high mobility so as to restore the compositional balance of the latter [11]. According to the NCAT categorization [12], petroleum-based rejuvenators include the types of paraffinic oils, aromatic extracts, and naphthenic oils. On the other hand, a variety of bio-oils have been evaluated in the literature with respect to their rejuvenating effects, including those derived from seeds, waste wood, waste cooking oil, and swine manure, to list a few [13,14,15,16]. The immediate impacts and particularly the long-term effects of rejuvenators differ depending on the material chemistry, which is in turn a function of their compositions and sources [17,18,19]. It has been demonstrated that effective applications in aged asphalts were able to lower the stiffness, increase the phase angle, and improve the resistance to fatigue and/or low-temperature cracking [15,20,21]. At the micro- and nano-scale, the effective use of rejuvenators in some cases seemed to be able to reverse the surface morphological changes induced by aging and to dissociate the asphaltene agglomerations [18,19]. Additionally, it is worth mentioning that rejuvenators do not chemically react with asphalts [13,21] and hence a reduction of the typically used infrared indices (e.g., for carbonyl and sulfoxide groups) as reported in some studies should be attributed to the physical diluting effect of rejuvenators. The same reason of no chemical reactions also emphasizes that rejuvenators cannot reverse the process of oxidation but instead they are aimed at reversing the impacts of aging on engineering properties and performance [9]. 

Plasticizer refers to a substance that is commonly added to polymers (e.g., plastics and rubber) to promote the flexibility and plasticity. The mechanism is associated with the good compatibility at the molecular level such that the plasticizer molecules are embedded between the polymer chains, thereby breaking down the interactions among the polymer molecules [22]. Recently, a variety of plasticizers have been introduced into the paving asphalts as modifiers and rejuvenators [23]. A cold resistant plasticizer, dioctyl adipate, has been shown to effectively decrease the glass transition temperature of a base asphalt and enhance the low-temperature crack resistance at the expense of reduced high-temperature performance [24]. Song et al. demonstrated that adding a certain amount of polyphosphoric acid into the asphalts modified with plasticizers (dioctyl phthalate, and trioctyl trimelliate) improved the stiffness and elastic recovery properties without sacrificing the low-temperature performance [25]. Jamal et al. reported that Cereclor (essentially a paraffin with a high percentage of chlorine) physically blended well with and significantly softened the aged asphalt [26]. Evaluation of the rejuvenated asphalt mixtures further showed that Cereclor substantially improved the fatigue performance while maintaining a satisfactory moisture resistance. Zhu et al. assessed the effect of a compound rejuvenator consisting of cotton oil and dibutyl phthalate on neat and polymer modified asphalts aged in the laboratory [27]. The results demonstrated pronounced benefits of the rejuvenator in restoring the stiffness and crack resistance. 

It has been well documented in the literature that different recycling agents may yield different rejuvenating effects [28]. Some additives serve only as a supplement to the solvent phase that is lost during aging; they do not engage in effective interactions with the asphalt molecules and have been considered as softeners [9,29]. In contrast, rejuvenators are expected to not only compensate the lost light fractions with adequate diffusion property but also be capable of dispersing and deagglomerating the asphaltenes by interacting with them [30].

Despite the limited studies up to the present, the existing findings have revealed an effective softening role of typical plasticizers and their excellent compatibility with asphalt materials. Some of them appear to be promising candidates for recycling oxidized asphalts but their rejuvenating effectiveness warrants further evaluation. In particular, the underlying mechanism has remained largely unclear at the molecular level, and whether they would function as rejuvenators or softeners remains to be investigated. The present study is aimed at evaluating the rejuvenating potential of two common plasticizers by rheological characterizations and to investigate the rejuvenating mechanisms by probing their interactions with the asphaltene molecules via molecular dynamics (MD) simulation.

## 2. Materials and Rheological Evaluation Methods

### 2.1. Materials

The asphalt binder used in this study was a 90# penetration grade petroleum asphalt, with a penetration of 84.0 (0.1 mm) at 25 °C, a softening point of 49.2 °C, and ductility of 142 cm at 15 °C. Laboratory aging was conducted using the Rolling Thin-Film Oven (RTFO) according to AASHTO T240 [31], followed by the pressurized aging vessel (PAV) at 90 °C under a pressure of 2.1 MPa for 20 h according to AASHTO R28 [32]. Two plasticizers were selected for investigation with respect to their rejuvenating potential: dibutyl phthalate (DBP) and tributyl citrate (TBC). The dosages used were 1%, 3%, and 5% for DBP and 1%, 2%, and 3% for TBC, by weight of the aged asphalt. 

DBP is a conventional phthalate ester widely used to manufacture plastics for daily life products, such as shower curtains, raincoats, food wraps, and vinyl fabrics, to name a few. It is acknowledged that DBP may pose potential risks to human health and its use in consumer products has been limited by regulations, but recent scientific evidences indicated that the risks are very low [33]. TBC is a citrate ester that provides a more environmentally friendly alternative to phthalates in applications, such as cosmetics, toys, pharmaceutical coatings, and food contact films. Selection of the two plasticizers was further based on considerations regarding the molecular structural differences to be described later.

### 2.2. Rheological Characterization

The effects of the plasticizers in restoring the rheology of the aged asphalt were investigated using the frequency sweep and linear amplitude sweep (LAS) tests in an Anton Paar MCR302 dynamic shear rheometer. Given the low test variability typically observed, two replicates were prepared for each test condition. 

The frequency sweep was conducted from 0.1 to 100 rad/s at a series of temperatures ranging from 55 to 0 °C. The strain was controlled at 0.1% for the material deformation within the linear viscoelastic region. Two sets of parallel-plate geometry were utilized: a diameter of 8 mm with a gap of 2 mm for temperatures below 35 °C and a diameter of 25 mm with a gap of 1 mm for above. The test data were processed to construct master curves of the shear modulus and phase angle to inspect the impacts of the plasticizers on linear viscoelastic characteristics. 

The LAS test was performed at an intermediate temperature of 20 °C and a fixed frequency of 10 Hz, using the diameter of 8 mm with a gap setup of 2 mm. The strain amplitude increased linearly from 0.1% to 30% over three different cycle numbers: 3000, 6000, and 9000. The data were analyzed using the viscoelastic continuum damage (VECD) model for evaluating the effects of the plasticizers on fatigue performance [3,34].

#### 2.2.1. Linear Viscoelastic Properties

The frequency sweep test data at different temperatures were shifted to establish a master curve for the shear modulus at a reference temperature. The Christensen–Andersen (CA) model was then used to fit the curve [35]:

(1) $$\left|G^{*}\right|=G_{g}{\left[1+{\left(\omega_{c}/\omega_{r}\right)}^{\left(\log2\right)/R}\right]}^{-R/\log2}$$

where |G^*^| denotes the dynamic shear modulus, i.e., magnitude of the complex shear modulus G^*^; *G_g_* is glassy modulus; *R* is rheological index; *ω_c_* is crossover angular frequency; and *ω_r_* is the reduced angular frequency that is related to the physical angular frequency by *ω_r_* = *ωa_T_*, where *a_T_* is the time-temperature shift factor. 

Additionally, based on the master curves for |G^*^| and phase angle, the Glover-Rowe (G-R) parameter can be determined. It was shown to be closely related with the ductility of asphalt measured at 15 °C and 1 cm/min [36,37], and has been widely used as a convenient indicator in assessing the crack resistance of (rejuvenated) asphalts [19,38]. This parameter is defined as

(2) $$\mathrm{G-R}=\frac{\left|G^{*}\right|\cos^{2}\delta }{\sin\delta }$$

where *δ* is phase angle, and note that both |G^*^| and *δ* are evaluated at 15 °C and 0.005 rad/s. 

#### 2.2.2. Fatigue Performance

The LAS test data were processed to provide damage characteristic relationship, Equation (3), failure criterion, Equation (4), and the predictive equation for fatigue life, Equation (7). For better understanding the model development and significance, refer to the work by Cao and Wang [3,34]. The following briefly lists the key relationships that constitute the model framework. 

The damage characteristic relationship is expressed as

(3) $$C=1-aS^{b}$$

where *C* is the normalized dynamic shear modulus measuring the material integrity, *S* is an internal variable indicating the damage intensity, and *a* and *b* are regression constants. 

The failure criterion reads

(4) $$W_{sum}^{R}=\lambda \cdot SE^{\mu }$$

with

(5) $$W_{sum}^{R}=\int_{0}^{N_{f}}\frac{1}{2}\mathrm{DMR}\cdot C_{i}{\left(\gamma_{i}\cdot \left|G_{\mathrm{LVE}}^{*}\right|\right)}^{2}dN$$

(6) $$SE=\left(\overset{N_{f}}{\underset{i=1}{\sum }}\gamma_{i}\right){\left|G_{\mathrm{LVE}}^{*}\right|}^{2}$$

where *W^R^_sum_* is accumulated pseudo-strain energy; *SE* stands for straining effort; DMR denotes dynamic modulus ratio representing the sample-to-sample variability; *γ_i_* is strain amplitude of the *i*-th test cycle; |G^*^_LVE_| is the linear viscoelastic shear modulus corresponding to the LAS test condition (temperature and frequency); *N_f_* is the number of cycles to failure identified at the peak of *C*^2^ × *N* × (1 − *C*) where *N* is the LAS cycle number; and *λ* and *μ* are regression constants.

The fatigue life predictive equation for a given strain level *γ*_0_ is written as

(7) $$\lambda {\left(N_{f}\right)}^{\mu -1}-\frac{1}{2}\gamma_{0}^{2-\mu }{\left|G_{\mathrm{LVE}}^{*}\right|}^{2-2\mu }\left[1-aM^{b}\frac{1}{1+b/k}{\left(N_{f}\right)}^{b/k}\right]=0$$

with the intermediate variables defined as

(8) $$M\equiv {\left[\frac{ab}{2}{\left(\gamma_{0}\cdot \left|G_{\mathrm{LVE}}^{*}\right|\right)}^{2}\right]}^{\alpha /k}{\left(kQ\right)}^{1/k}$$

and

(9) $$\{\begin{array}{c} Q\equiv \int_{0}^{2\pi /\omega_{r}}\sin^{2\alpha }\left(\omega_{r}\xi \right)d\xi \\ k\equiv 1+\alpha -b\alpha \end{array}$$

where *α* is damage evolution rate identified from the |G^*^| master curve, and *ξ* is reduced time.

The *N_f_* versus *γ*_0_ data pairs are then plotted in the double-logarithmic space to yield the so-called failure envelope, which provides a direct measure and convenient comparison of the fatigue resistance for asphalt binders.

## 3. Molecular Dynamics Simulation Approach

**Figure 1:**
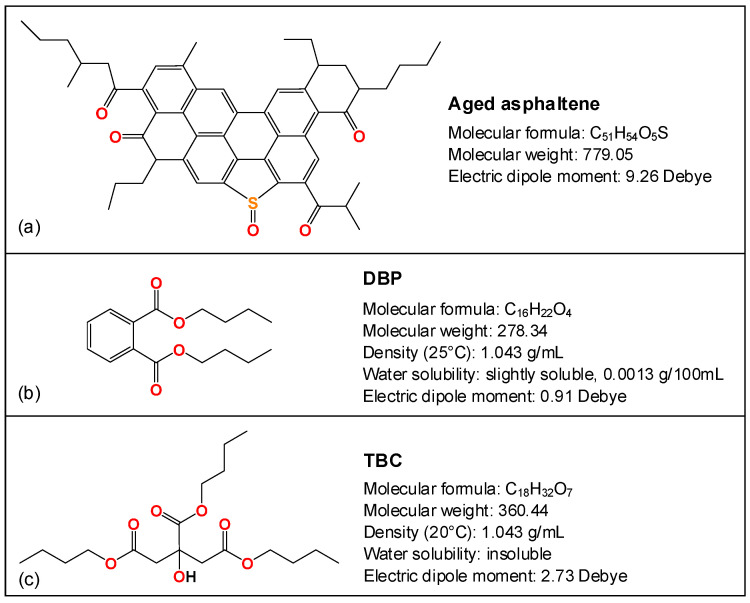
Molecular structures of (**a**) the asphaltene and (**b**,**c**) plasticizers used in simulation.

Compared to other components in the asphalt system, asphaltenes are known to have a higher tendency to self-associate forming nano- and even macro-agglomerates. MD simulations have been performed to visualize the self-interactions among the asphaltenes and examine the dissociating potential of various rejuvenators in a solvent environment provided by heptane and/or toluene [39,40]. The asphaltene-thiophene proposed by Li and Greenfield based on Mullins’ work was selected as the model molecule for the asphaltenes [41,42]. Since ketones and sulfoxides are the major oxidation products formed at the benzyl carbons and alkyl/aryl sulfides, respectively, the virgin structure was modified accordingly to represent the oxidized counterpart, as shown in Figure 1a.

The molecular structures of the two plasticizers, Figure 1b,c, feature the presence of polar ester groups that would help to promote their compatibility and interaction with asphalt molecules [43]. TBC contains three ester groups and an additional hydroxyl group that may form hydrogen bonds with oxidized asphalts. In contrast, DBP has only two ester groups and thus a much lower polarity, with an electric dipole moment of 0.91 Debye (compared to 2.73 Debye for TBC). Nevertheless, the presence of the benzene ring in DBP is expected to cause aromatic-aromatic interactions (π-stacking) with the polyaromatic structures abundant in asphaltenes [40]. Despite the environmental concerns related to the use of DBP, this phthalate ester was included mainly for the purpose of elucidating how the structural differences in the two plasticizers would impact their interactions with the asphaltenes. The finding is expected to assist in the selection and development of rejuvenators.

**Figure 2:**
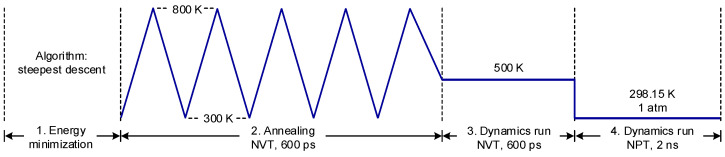
The four-step process used to equilibrate the molecular systems.

The simulation was carried out using the Gromacs package (version 2021.2) [44] in conjunction with the general Amber force field (GAFF) [45] that has been shown to provide reasonable predictions for some physical properties of asphalts [46]. As the reference system, a total of 16 oxidized asphaltene molecules and 500 heptane molecules were randomly placed in a cubic box with a dimension of 7.0 × 7.0 × 7.0 nm^3^. In the rejuvenated systems, another 16 DBP or TBC molecules were added into the reference system. These initial systems were then subjected to a four-step process to reach equilibration, as illustrated in Figure 2:

Step 1: Energy minimization, using the steepest descent algorithm with the maximum number of steps set to 50,000;
Step 2: Annealing, following a triangular temperature profile covering the range from 300 to 800 K. The ending temperature was set at 500 K, the same temperature used for the subsequent dynamics run. The canonical NVT ensemble (constant value for the number of particles, volume, and temperature) was used for this stage;
Step 3: Dynamics run, for a period of 600 ps under 500 K using the NVT ensemble;
Step 4: Dynamics run, for a period of 2 ns to equilibrate the system under the room temperature and atmospheric pressure using the NPT ensemble (constant value for the number of particles, pressure, and temperature). The densities of all the molecular systems evaluated were found to stabilize within 600 ps.

Subsequent to the equilibration, a further NVT ensemble (298.15 K) was applied to each system for 20 ns to generate the steady-state data, for analyses using the mean squared displacement, radial distribution function, aggregation number, and order parameter. For all simulation stages, the velocity rescaling algorithm was adopted for temperature coupling, and the Berendsen barostat was employed for the NPT ensemble. The short-range cut-off distances for both the electrostatic and van der Walls interactions were set at 2.0 nm. For the long-range electrostatic interactions, the particle-particle-particle-mesh algorithm was applied.

## 4. Results and Discussions

This section presents the results from the rheological characterization and MD simulation. Discussions are provided regarding the rejuvenating potential of the two plasticizers and also with respect to how the structural differences of the plasticizer molecules would influence their interactions with the asphaltenes.

### 4.1. Rheological Performance

#### 4.1.1. Linear Viscoelastic Characteristics

**Figure 3:**
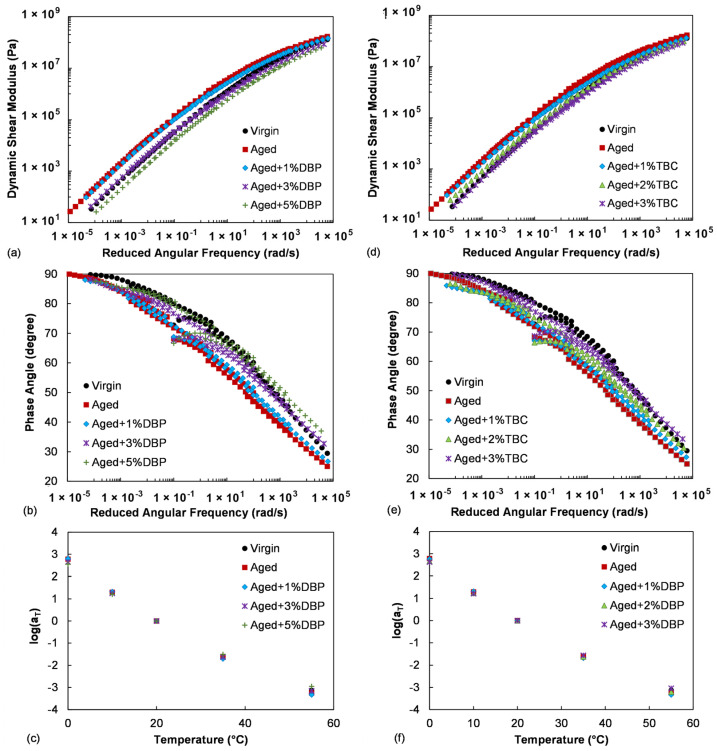
Master curves of the dynamic shear modulus and phase angle and the time-temperature shift factors for evaluating the effects of (**a**–**c**) DBP, and (**d**–**f**) TBC.

Figure 3 provides the frequency sweep test results at a reference temperature of 20 °C for the aged asphalt with different percentages of the plasticizers. The results for the virgin binder were also included for comparison. Both DBP and TBC demonstrated effective rejuvenating effects as they consistently lowered the stiffness, while restoring the phase angle with increase in the dosage. Use of 5% DBP was seen to over soften the aged asphalt, as indicated by the resulting modulus master curve lying under that of the virgin across the full range of reduced frequency in Figure 3a.

It is interesting to note that as compared to the virgin, the two plasticizers with a blending dosage of 3% were able to bring the stiffness of the aged asphalt down to a similar level in the low reduced frequency range (corresponding to high temperature), while providing similar or slightly lower phase angles. Meanwhile, in the high reduced frequency range (corresponding to low temperature), the rejuvenated stiffness was lower than that of the virgin but the phase angles were similar or slightly higher. In general, at high temperatures a higher stiffness associated with a lower phase angle (meaning a higher elasticity) is desired for resisting permanent/plastic deformation. At low temperatures a lower stiffness with a higher phase angle (suggesting a higher capability of stress relaxation) is preferred for resisting cracking. On the basis of these concepts, with an appropriate blending dosage into the aged asphalt, the two plasticizers appeared to be able to restore the low-temperature performance without sacrificing the high-temperature rutting resistance as compared to the virgin. It is worth mentioning that both DBP and TBC are low-temperature resistant plasticizers and have been exploited to improve the low-temperature flexibility of polymer materials in the industry [47]. Additionally, for the asphalt evaluated in this study, the laboratory aging treatment and use of the plasticizers did not have significant impacts on the time-temperature shift factors.

**Figure 4:**
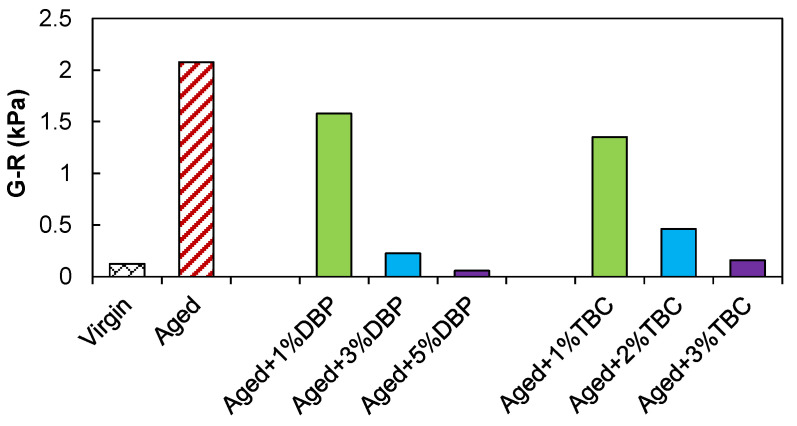
The G-R parameter results.

Figure 4 presents the G-R parameters for the asphalt binders. As a lower G-R value corresponds to a higher ductility that is desired for crack resistance, the two plasticizers demonstrated pronounced benefits in improving the cracking performance of the aged asphalt. Incorporation of 5% DBP was able to render the aged binder even more crack resistant than the virgin. On the other hand, a comparison between the two plasticizers at the same dosages of 1% and 3% suggested that TBC was more efficient in reducing G-R.

#### 4.1.2. Fatigue Performance

**Figure 5:**
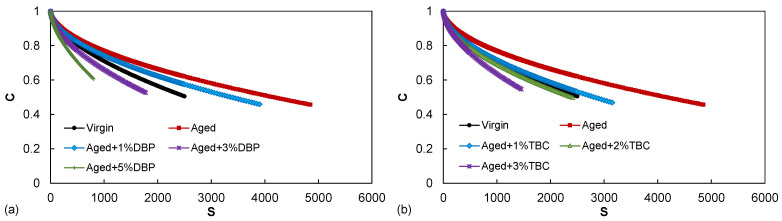
The damage characteristic relationships for evaluating the effects of (**a**) DBP, and (**b**) TBC.

The LAS test results were processed to first obtain the damage characteristic relationships, as shown in Figure 5. The *C*(*S*) function essentially prescribes the path along which a linear viscoelastic material follows, in the sense that the material loses the structural integrity as a result of damage evolution in the form of microcracking [2]. In general, the *C*(*S*) curves are positioned from top to bottom in the decreasing order of material stiffness (at the intact state) due to its involvement in the calculation of *S* [3]. For this reason, the position arrangement of the *C*(*S*) curves cannot be simply used to evaluate the fatigue performance. Rather, the relationships need to be further utilized to perform fatigue simulations using Equation (7).

**Figure 6:**
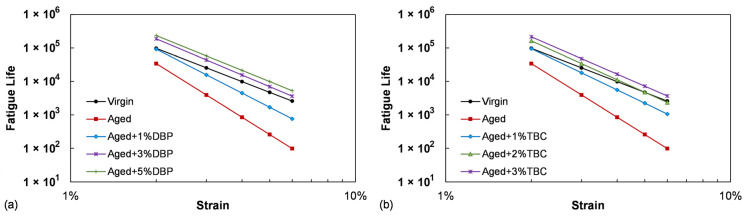
The predicted fatigue performance for evaluating the effects of (**a**) DBP, and (**b**) TBC.

Figure 6 presents the obtained failure envelopes (relationships between the predicted fatigue life and the strain amplitude) for the asphalt binders. Use of both plasticizers substantially enhanced the fatigue resistance by shifting the envelopes upward, which is consistent with the observations based on the G-R parameter. Incorporation of 1% DBP and TBC into the aged asphalt yielded similar fatigue lives at the strain level of 2%, comparable to that of the virgin binder. Use of 3% and beyond led to better performance than the virgin for all the strain conditions in the case of both plasticizers. A close inspection further indicates that TBC again demonstrated a slightly higher efficiency in restoring the fatigue performance at the same dosages compared to DBP.

A synthesis of the previous observations based on the master curves of modulus and phase angle, the G-R parameter as well as the fatigue performance suggests that in order to determine the optimum dosages, 3% appeared to be a proper starting point for both plasticizers. This incorporation percentage is much lower than those typically required for conventional rejuvenators but would increase with the severity of aging [19]. The rejuvenating effectiveness and the optimum dosages of the plasticizers should be further ascertained in a systematic experimental program that may involve performance evaluations related to rutting, low-temperature cracking, fatigue, moisture susceptibility, and perhaps even the long-term performance of the rejuvenated asphalts [29,48]. Depending on the findings, additional additives, such as anti-stripping and anti-oxidant agents, may be considered whenever necessary [49,50].

### 4.2. MD Simulation Results

The rheological performance of the two plasticizers in terms of restoring the stiffness, phase angle, and crack resistance of the aged asphalt seemed to suggest that they were indeed rejuvenating instead of simply softening the asphalt [9,29]. In order to further corroborate this hypothesis, the MD simulation was necessitated to visualize the molecular interactions between the rejuvenators and the oxidized asphaltenes. 

#### 4.2.1. Mean Squared Displacement

The mean squared displacement (MSD) provides a time-dependent measure of the spatial extent that is “explored” by the asphaltene molecules due to diffusion, defined as

(10) $$\mathrm{MSD}\left(t\right)=\frac{1}{N}\overset{N}{\underset{i=1}{\sum }}{\left|r_{i}\left(t\right)-r_{i}\left(0\right)\right|}^{2}$$

where *N* is the total number of particles to be averaged, and ***r****_i_*(0) and ***r****_i_*(*t*) denote the position vectors of the *i*-th particle at time 0 and *t*, respectively.

**Figure 7:**
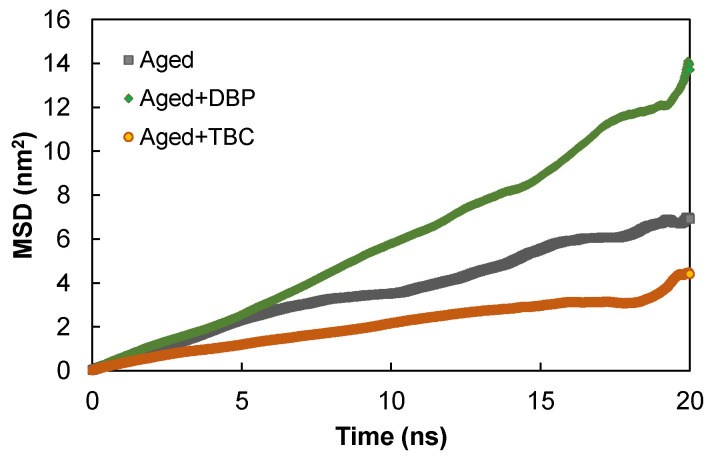
The time histories of the mean squared displacement at 298.15 K.

**Figure 8:**
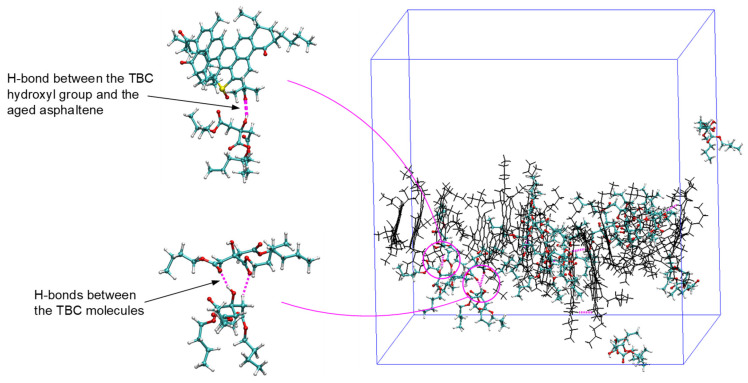
Snapshot of the simulation box consisting of aged asphaltenes, TBC, and heptane (not shown).

Figure 7 provides the time histories of MSD for the aged asphaltenes with and without the plasticizers equilibrated at the normal condition of 298.15 K and atmospheric pressure. Introduction of DBP promoted the mobility of the aged molecules, as evidenced by the longer distances travelled at a given time, especially at times beyond 5 ns. On the contrary, TBC appeared to slow down the diffusion of the aged asphaltenes, which can be ascribed to its higher polarity than DBP. The higher molecular polarity of TBC due to the presence of three ester groups were expected to result in stronger interactions with the asphaltenes [43]. Further, the hydroxyl group provided additional contributions by forming hydrogen bonds between TBC and asphaltenes and also among the TBC molecules themselves (as per the typical distance and angle criteria of 3.5 Å and 30°, respectively), as shown in Figure 8. These combined factors created spatial hindrance in the random movement of the asphaltenes at the room temperature condition. No hydrogen bonding was noted between DBP and asphaltenes due to the absence of hydrogen donors.

**Figure 9:**
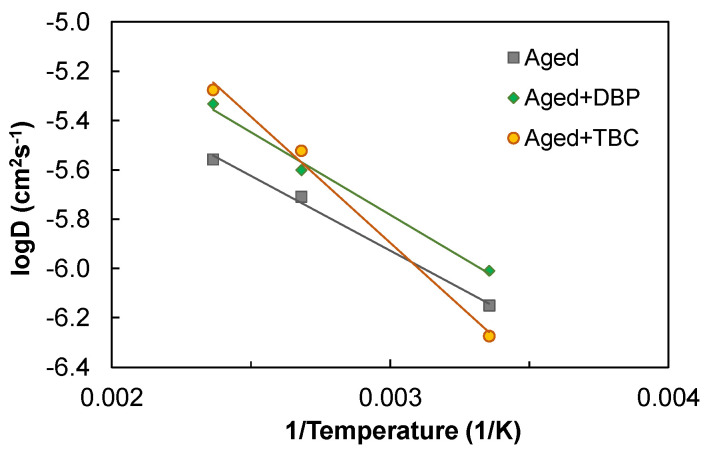
The temperature dependence of the diffusion coefficient and the Arrhenius fits.

Since diffusion is a physical property that is highly promoted by temperature rise, it was of interest to examine and compare the diffusion behaviors at higher temperatures. For this purpose, each molecular system was equilibrated at higher temperatures of 373.15 K and 423.15 K, by revising accordingly the coupling temperature in the NPT ensemble of the four-step process, as shown earlier in Figure 2. Similarly, the MSD histories were obtained using the data produced from the subsequent NVT ensemble, and the slope was employed to determine the diffusion coefficient by a factor of 1/6 according to the Einstein relation [51]. Figure 9 illustrates the dependence of the diffusion coefficient of asphaltenes on temperature and also the Arrhenius fits [52]

(11) $$D=D_{0}e^{-\frac{E_{a}}{RT}}$$

where *D* denotes the diffusion coefficient, *E_a_* is activation energy, *R* is the universal gas constant (8.314 J/mol/K), *T* is temperature, and *D*_0_ the diffusion pre-factor.

In general, the Arrhenius relationship reasonably described the temperature-dependent diffusion of asphaltenes in all the molecular systems. The addition of DBP increased consistently the mobility of asphaltenes at all temperatures. It is interesting to note that despite the slowest diffusion at 298.15 K, use of TBC led to the highest *D*-values at higher temperatures. It is indicated that the temperature increase allowed the asphaltenes to overcome and escape from the energy barrier created by their strong interactions with TBC.

#### 4.2.2. Radial Distribution Function

The radial distribution function (RDF), *g*(*r*) is defined as the ratio of the local number density of particles at a distance *r* away from a reference particle, *ρ*(*r*), to the average (bulk) number density of particles in the system, *ρ* [53]

(12) $$g\left(r\right)=\frac{\rho \left(r\right)}{\rho }$$

The RDF itself is not a probability distribution, but it provides the most probable distances between the asphaltene pairs with and without the interference of plasticizer molecules. In general, *g*(*r*) is calculated as an average over a thermodynamic ensemble. Integration of *g*(*r*) on spherical shells over the radius *r* gives the coordination number *N*(*r*), or the number of neighboring particles

(13) $$N\left(r\right)=\int_{0}^{r}\rho g\left(r\right)4\pi r^{2}dr$$

**Figure 10:**
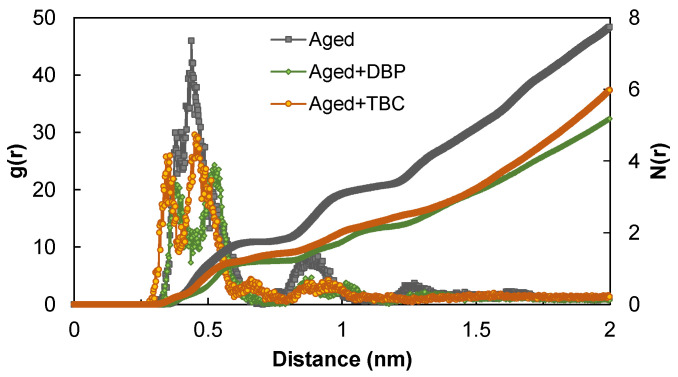
The radial distribution functions and the coordination numbers.

**Figure 11:**
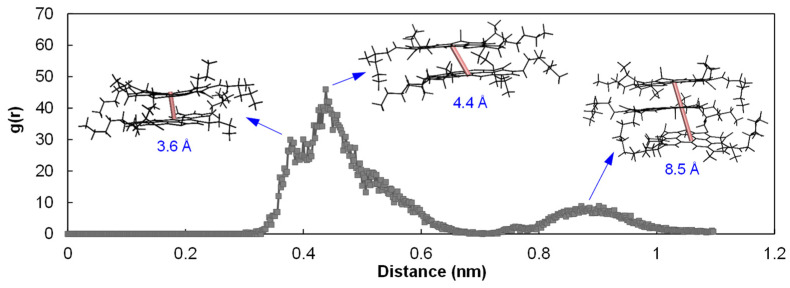
Configurations of the asphaltene agglomerates for different ranges of COM distances.

Figure 10 gives the RDF and coordination number results for the three molecular systems. For all the cases, two closely separated peaks were identified within the distance range below 6 Å, representing the (offset) parallel stacking configuration of the asphaltenes, as shown in Figure 11. Specifically, use of TBC slightly shifted the first peak toward the lower distance by approximately 0.4 Å, and DBP shifted the second peak toward the longer distance roughly by 1.0 Å. Both plasticizers were able to reduce the RDF peaks at the ranges within 6 Å as well as between 8 and 10 Å, as also reflected in the coordination number curves. On average, the plasticizers dispersed and dissociated the asphaltenes by decreasing the number of surrounding asphaltenes with respect to a reference.

It was worth mentioning the agglomerate configuration for the RDF peak appearing between 8 and 10 Å in the aged system. Even though the literature reported that it was in general due to T-shaped stacking [54], our simulation demonstrated that this peak was mostly attributed to the distance between the two outer monomers in a parallel stacking trimer configuration, as shown in Figure 11.

#### 4.2.3. Aggregation Number

**Figure 12:**
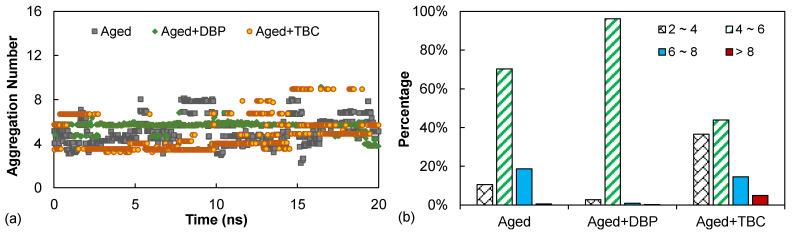
The aggregation number results: (**a**) time histories, and (**b**) distributions.

The aggregation number provides a direct quantification for the degree of self-association of the asphaltene molecules, defined as [40]

(14) $$g_{z}=\underset{i}{\sum }n_{i}g_{i}^{3}/\underset{i}{\sum }n_{i}g_{i}^{2}$$

where *g_z_* is the *z*-average aggregation number, and *n_i_* is the number of agglomerates containing *g_i_* monomers. A higher *g_z_* suggests a higher degree of self-association of asphaltenes. For the systems considered in this study with a total of 16 asphaltene molecules, *g_z_* would vary between 1 (the asphaltenes all separate from each other, not forming any agglomerates) and 16 (all the molecules agglomerate to form a single cluster). Figure 12 provides the time histories and distributions of the aggregation number over the 20 ns NVT ensemble dynamics run.

As illustrated in Figure 12a, none of the molecular systems attained a *g_z_*-value of 1 or 2, indicating that the self-association of asphaltenes always occurred to a greater or lesser extent. According to the distributions shown in Figure 12b, the asphaltene aggregation state in the aged system was dominated by aggregation numbers below 8 for which roughly 80% of the states were characterized by small (*g_z_* < 4) and intermediate agglomerates (4 < *g_z_* < 6). The addition of DBP effectively reduced the number of moderately large (6 < *g_z_* < 8) agglomerates. This effect can be attributed to the benzene ring present in the DBP molecule that interacted with the asphaltene aromatic cores, limiting their growth into larger agglomerates [40]. Use of TBC appeared to encourage the formation of large agglomerates (*g_z_* > 8), corresponding to the slight shift of the first RDF peak to shorter distances, as previously noted in Figure 10. This observation was presumably due to the aforementioned strong interactions between TBC and asphaltenes as well as among the TBC molecules themselves. Nevertheless, this adverse impact appeared to be insignificant. Compared to the aged system, the percentage of *g_z_* < 6 was approximately the same, but TBC demonstrated a significant deagglomerating effect in increasing the share of small associations.

#### 4.2.4. Order Parameter

An order parameter, as the name suggests, measures the degree of order across the system. Its definition varies depending on the specific phenomenon under consideration and has been used frequently in dealing with phase transition and magnetization [55,56]. It is recognized that the previous three MD parameters describe physical characteristics of the asphaltenes all based on the particle-wise considerations. The interactions among the asphaltenes in general tend to form stacking and T-shape configurations [39], which suggests that the molecular orientation should also be taken into account as an important complement in characterizing the aggregation behavior.

In this study, the orientational order parameter was employed to investigate the orientational distribution of the asphaltene molecules, defined as

(15) $$\varphi =\frac{1}{2}\left(3\langle \cos^{2}\theta \rangle -1\right)$$

where *φ* denotes the order parameter, *θ* is the inclination angle of a specific molecule with respect to a reference direction, and 〈〉 denotes average over all the molecules. For any system snapshot, the best-fit plane for each asphaltene molecule was first identified using the coordinates of the aromatic carbons. The angles formed between the planes and the *z*-axis were then averaged to obtain the reference orientation, which was then substituted into Equation (15) to determine the order parameter for each frame. According to the definition, a set of perfectly aligned asphaltenes corresponds to an order parameter of unity, while increase in the randomness of the molecular orientations would reduce the order parameter. 

**Figure 13:**
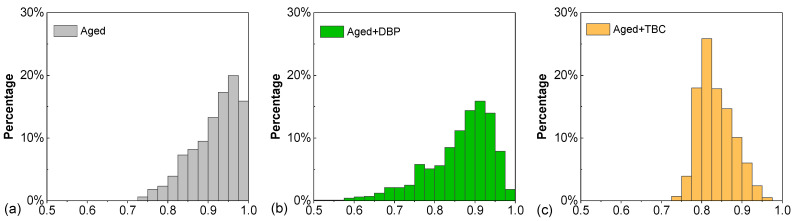
Distributions of the order parameters for (**a**) aged asphalt, (**b**) aged asphalt with DBP, and (**c**) aged asphalt with TBC.

Figure 13 presents the distributions of the order parameter for the three molecular systems. The asphaltenes in the aged system provided a distribution skewed toward the unity, which evidenced that the molecules tended to be highly ordered in terms of orientation, promoting the formation of parallel stacking agglomerates. Introduction of DBP shifted the distribution toward lower *φ*-values and in the meanwhile widened the spread of the order parameter. Recall in Figure 12b that DBP was able to dissociate the aged asphaltenes into intermediate agglomerates, which had a higher degree of freedom to assume different orientations, hence the increased range of *φ*. TBC had a negligible impact on the range of *φ* but exhibited a higher effectiveness in shifting the distribution toward the lower end, i.e., reducing the degree of orientational orderliness. The strong molecular polarity and the hydroxyl group allowed TBC to be more capable of interfering with asphaltenes and promoting the randomness of their orientations, thereby hindering the parallel stacking.

## 5. Conclusions

This study selected traditional and environmentally friendly plasticizers based on the molecular structural discrepancy and investigated their potential in rejuvenating the oxidatively aged paving asphalts from the rheological and molecular perspectives. The following summarizes the key conclusions drawn based on the findings:

- The two plasticizers were both highly effective in softening the aged asphalt. As interred from their impacts on the dynamic shear modulus and phase angle master curves, incorporation of appropriate dosages of DBP or TBC were able to retore the low-temperature performance to a level comparable to the virgin asphalt without sacrificing the stiffness and elasticity properties required at high temperatures;
- Increase in the blending dosages of both plasticizers consistently improved the fatigue resistance of the aged asphalt. At the same dosages of 1% and 3%, TBC demonstrated a slightly higher effectiveness than DBP in restoring the fatigue performance by providing lower G-R values and higher fatigue lives;
- Compared to DBP, TBC provided stronger interactions with the aged asphaltenes due to the presence of three polar ester groups and a hydroxyl group that formed hydrogen bonds with the latter. The stronger interactions hindered the mobility of asphaltenes at the room temperature condition but temperature rise enabled the asphaltenes to overcome the energy trap. At higher temperatures, TBC promoted the mobility by yielding the highest diffusion coefficients of the asphaltenes;
- Analyses based on RDF and order parameter indicated the deagglomerating effects of both plasticizers. They dispersed and dissociated the asphaltenes by decreasing the number of molecules surrounding a reference and by promoting the orientational randomness of asphaltene molecules so as to weaken the formation and growth of parallel stacking agglomerates;
- The benzene ring structure present in DBP allowed aromatic interactions with the cores of asphaltenes, limiting their growth into larger agglomerates; the resulting aggregation state was dominated by intermediate-sized agglomerates according to the aggregation number results. Owing to its high polarity and hydroxyl group, TBC exhibited a slightly better deagglomerating effect as it yielded a higher percentage of small agglomerates and on average a lower orientational orderliness of the asphaltenes. This observation helped to shed light on the higher rejuvenating effectiveness of TBC as noted in the rheological performance.

The rheological characterizations suggested that the two plasticizers were rejuvenating, not simply softening the aged asphalt, while the MD simulation indicated that the rejuvenating effects were ascribed to the plasticizer molecules being able to disperse and dissociate the asphaltenes. Future study is needed to assess the rejuvenating effects from different performance perspectives and to optimize the incorporation dosages. Additional effort may be devoted to the long-term rejuvenating effectiveness of the plasticizers, considering the possible oxidation and volatilization of the rejuvenator molecules.

## Data Availability

The data that support the findings also form part of an on-going study and will be available from the corresponding author on request.
